# National Surveillance of Human Ehrlichiosis Caused by *Ehrlichia ewingii*, United States, 2013–2021

**DOI:** 10.3201/eid3102.240279

**Published:** 2025-02

**Authors:** Sydney N. Adams, Nicolette C. Bestul, Kimberly N. Calloway, Gilbert J. Kersh, Johanna S. Salzer

**Affiliations:** Oak Ridge Institute for Science and Education, Oak Ridge, Tennessee, USA (S.N. Adams); Centers for Disease Control and Prevention, Atlanta, Georgia, USA (S.N. Adams, N.C. Bestul, K.N. Calloway, G.J. Kersh, J.S. Salzer)

**Keywords:** ehrlichiosis, *Ehrlichia ewingii*, ticks, surveillance, epidemiology, bacteria, vector-borne infections, zoonoses, United States

## Abstract

Human ehrlichiosis is a potentially fatal tickborne disease caused by 3 species: *Ehrlichia chaffeensis*, *E. ewingii*, and *E. muris eauclairensis*. In the United States, 234 confirmed cases of *E. ewingii* ehrlichiosis were reported to the Centers for Disease Control and Prevention through the National Notifiable Diseases Surveillance System during 2013–2021; average annual incidence was 0.08 cases/1 million population. *E. ewingii* ehrlichiosis was reported more commonly among older, White, non-Hispanic, and male patients. Incidence and case counts generally increased yearly, except for 2020 and 2021. The highest number of cases were reported from Missouri and Arkansas. We report the geographic expansion of *E. ewingii* ehrlichiosis and the continued public health challenge of clarifying clinical manifestations of this infection. Clinician education will be essential to implement molecular assays to properly diagnose *E. ewingii* infection in patients and gain a better understanding of the epidemiology of this emerging disease.

In the United States, human ehrlichioses are potentially fatal tickborne rickettsial diseases caused by the intracellular bacterium belonging to the genus *Ehrlichia* (*1*). Three species of *Ehrlichia* are primarily associated with human disease: *E. chaffeensis*, *E. ewingii*, and *E. muris eauclairensis*. Most ehrlichiosis cases reported to the Centers for Disease Control and Prevention (CDC) have been caused by *E. chaffeensis*. Although *E. ewingii* ehrlichiosis cases are reported less frequently to CDC, the numbers of reported cases have increased overall since 2008 (*2*).

The clinical severity of ehrlichiosis depends upon the *Ehrlichia* species causing infection and underlying risk factors. In general, *E. ewingii* infections have been considered milder than infections caused by *E. chaffeensis* (*3*). Persons with ehrlichiosis who are immunocompromised, especially those who have HIV, are receiving cancer treatments, or had organ transplants, are at the highest risk for severe outcomes (*3*–*9*). Only 1 fatal case of *E. ewingii* ehrlichiosis was reported to CDC in 2018.

Various methods can be used to diagnose ehrlichiosis in patients. However, to differentiate between *E. ewingii* and other *Ehrlichia* spp., PCR or other molecular methods are necessary. Serologic testing cannot differentiate among *Ehrlichia* spp. responsible for human infections (*10*,*11*).

Ehrlichiosis was first made nationally notifiable in the United States in 1999 by the Council of State and Territorial Epidemiologists (CSTE) (*12*). However, the case definition at that time did not differentiate between infections caused by *Ehrlichia* spp. or *Anaplasma* spp. and did not permit species-specific reporting. In 2008, the CSTE case definition was revised to distinguish between ehrlichiosis and anaplasmosis and to include the following categories for ehrlichiosis: *E. chaffeensis* infection (formerly human monocytic ehrlichiosis); *E. ewingii* infection (formerly ehrlichiosis [unspecified or other agent]); and ehrlichiosis/anaplasmosis, human, undetermined. Because *E. muris eauclairensis* was discovered after the adoption of the 2008 case definition, it was not included in the categories of ehrlichiosis until a revised case definition was established in 2024.

Although *E. ewingii* ehrlichiosis is a nationally notifiable condition, each jurisdiction in the United States determines what conditions are reportable in their respective area through the local legislative process. Therefore*, E. ewingii* ehrlichiosis is not a reportable infection in some jurisdictions. Here, we describe the passive surveillance of *E. ewingii* ehrlichiosis reported to the National Notifiable Diseases Surveillance System (NNDSS) in the United States during 2013–2021.

## Methods

State and territorial health departments voluntarily submit confirmed and probable cases of ehrlichiosis to CDC through NNDSS. During 2013–2021, *E. ewingii* infection was reportable in 44 US states and the District of Columbia. Variables transmitted to the NNDSS are sex, age, race, ethnicity, event date, county and state of residence, import status (outside county, state, or country), case status, and whether the case is associated with an outbreak. Cases were defined by using the 2008 CSTE case definition (*12*). The event date is the earliest date associated with the case, which can be the symptom onset date, diagnosis date, laboratory test date, or the date the case was reported to the county or state health department. We included in the analysis all confirmed *E. ewingii* ehrlichiosis cases reported to CDC through NNDSS until November 1, 2023, that had event dates during January 1, 2013–December 31, 2021.

We used NNDSS data and population estimates from the US Census Bureau to calculate national-, state-, county-, and age-specific ehrlichiosis incidence. We calculated each year’s cumulative incidence and then averaged those data over the study period to produce the average annual incidence; we did not include states where *E. ewingii* ehrlichiosis was not a notifiable condition. *E. ewingii* infection was not considered a notifiable disease during some years in Alaska (2013–2021), Colorado (2013–2021), Connecticut (2015–2021), District of Columbia (2013), Hawaii (2013–2021), Idaho (2013–2021), Iowa (2013–2015), and New Mexico (2013–2021). We included cases reported as infections acquired outside of the state of residence or outside of the United States in the overall case counts but did not include those cases in state or county level counts or incidence. We did not include infections acquired outside of the United States in the national incidence calculations. We did not include missing values in the denominator for frequencies and rates, unless otherwise noted. We analyzed data by using SAS version 9.4 (SAS Institute Inc.).

## Results

**Figure 1:**
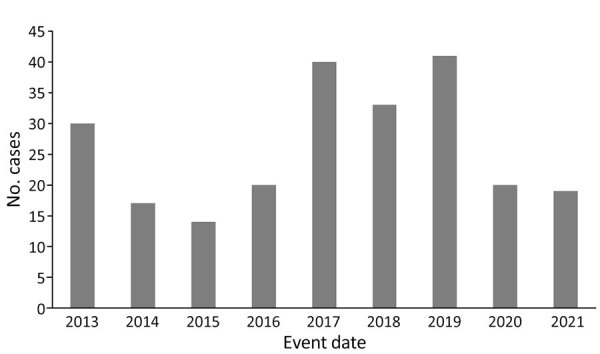
Annual number of human ehrlichiosis cases caused by *Ehrlichia ewingii* according to event date, United States, 2013–2021. Confirmed cases of *E. ewingii* ehrlichiosis were reported to the Centers of Disease Control and Prevention through the National Notifiable Disease Surveillance System. Event dates were the earliest date associated with the case, which could be the symptom onset date, diagnosis date, laboratory test date, or the date the case was reported to the county or state health department.

**Figure 2:**
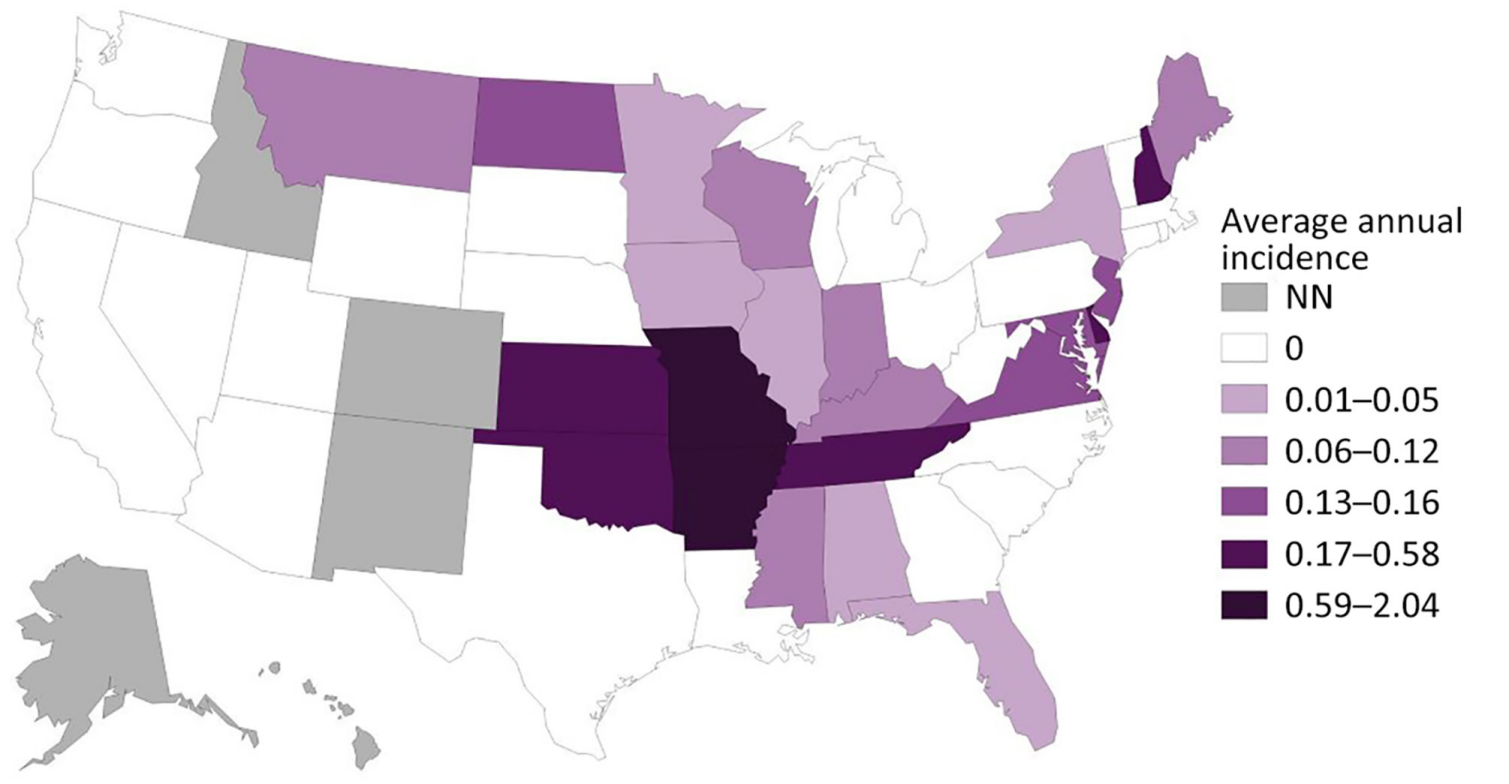
Average annual incidence (cases/1 million persons) of human ehrlichiosis caused by *Ehrlichia ewingii*, United States, 2013–2021. Confirmed cases of *E. ewingii* ehrlichiosis were reported to the Centers of Disease Control and Prevention through the National Notifiable Disease Surveillance System. *E. ewingii* ehrlichiosis was not a notifiable disease in some states. NN, not notifiable (during the entire study period).

A total of 245 ehrlichiosis cases caused by *E. ewingii* with an event date during 2013–2021 were reported to NNDSS. Of those cases, 234 (96%) were reported as confirmed (Table 1). Eleven cases were reported as probable; we did not include those cases in the analysis because we were unable to apply CSTE case definitions post hoc. Case counts generally increased over the study period, peaking in 2019 (Figure 1). The average annual incidence for the study period was 0.08 cases/1 million population. The annual incidence during the study period varied but peaked at 0.13 cases/1 million population in 2017 and in 2019. States that reported the most cases were Missouri (n = 112), Arkansas (n = 22), Kansas (n = 11), New Jersey (n = 11), Tennessee (n = 10), Virginia (n = 10), Maryland (n = 7), Oklahoma (n = 6), Delaware (n = 5), and Kentucky (n = 5) (Table 2). Those 10 states accounted for 86% (n = 199) of all reported *E. ewingii* infection cases in the United States; Missouri alone accounted for 48% of all cases reported and had the highest incidence (Figure 2). Overall, 24 jurisdictions reported >1 case of *E. ewingii* ehrlichiosis, and 15 (63%) states reported cases for the first time during 2013–2021 (Table 2). Kansas and Virginia both had low case counts but had the largest increases in cases during the study period; Kansas had a 3.2-fold (n = 11) increase, and Virginia had a 2-fold (n = 11) increase in reported incidence.

**Table 1:** Demographic characteristics of patients in study of national surveillance of human ehrlichiosis caused by *Ehrlichia ewingii*, United States, 2013–2021*

Characteristics	No. patients (%)	Incidence†
Total no. patients	234	NA
Sex		
M	149 (63.7)	0.14
F	84 (35.9)	0.06
Unknown	1 (0.4)	NA
Race‡		
White	163 (69.7)	NA
Black	2 (0.9)	NA
American Indian/Alaska Native	0	NA
Asian/Pacific Islander	3 (1.3)	NA
Other	7 (3.0)	NA
Missing	2 (0.9)	NA
Unknown	57 (24.4)	NA
Ethnicity‡		
Hispanic	3 (1.3)	NA
Non-Hispanic	193 (82.5)	NA
Unknown	38 (16.2)	NA
Age, y		
<10	0	NA
10–19	6 (2.6)	0.08
20–29	8 (3.4)	0.08
30–39	18 (7.7)	0.12
40–49	25 (10.7)	0.09
50–59	62 (26.5)	0.17
60–69	54 (23.1)	0.13
>70	61 (26.1)	0.13
Unknown	0	NA
Median age, y (IQR)	59 (50–70)	NA

*Values are no. (%) except as indicated. Confirmed cases of ehrlichiosis caused by *Ehrlichia ewingii* were reported to the US National Notifiable Disease Surveillance System. IQR, interquartile range, NA, not applicable. †Average incidence over the reporting period. ‡Because of large proportions of missing data, incidence was not calculated for race or ethnicity.

**Table 2:** Ehrlichiosis cases caused by *Ehrlichia ewingii* reported to the National Notifiable Disease Surveillance System, United States, during 2013–2021 and 2008–2012*

State or region	No. cases, 2013–2021	No. cases, 2008–2012
Alabama	1	0
Alaska	NN	NN
Arizona	0	0
Arkansas	22	0
California	0	0
Colorado	NN	NN
Connecticut†	0	0
Delaware	5	5
District of Columbia†	0	NN
Florida	3	0
Georgia	0	1
Hawaii	NN	NN
Idaho	NN	NN
Illinois	4	1
Indiana	4	0
Iowa†	1	NN
Kansas	11	1
Kentucky	5	0
Louisiana	0	1
Maine	1	0
Maryland	7	2
Massachusetts	0	0
Michigan	0	0
Minnesota	2	2
Mississippi	2	0
Missouri	112	33
Montana	1	NN
Nebraska	0	0
Nevada	0	0
New Hampshire	2	0
New Jersey	11	0
New Mexico	NN	NN
New York City	2	0
New York	5	0
North Carolina	0	0
North Dakota	1	0
Ohio	0	1
Oklahoma	6	0
Oregon	0	0
Pennsylvania	0	0
Rhode Island	0	0
South Carolina	0	1
South Dakota	0	0
Tennessee	10	5
Texas	0	0
Utah	0	0
Vermont	0	0
Virginia	10	2
Washington	0	0
West Virginia	0	0
Wisconsin	3	0
Wyoming	0	0

*Ehrlichiosis caused by *E. ewingii* is not a notifiable disease in some states. NN, not notifiable (during the entire study period). †Ehrlichiosis caused by *E. ewingii* was not notifiable in the jurisdiction for part of the reporting period (Connecticut 2015–2021, District of Colombia 2013, Iowa 2013–2015). Data represent the years when the condition was notifiable in the jurisdiction.

More cases of *E. ewingii* ehrlichiosis were reported in male (n = 149 [64%]) than female (n = 84 [36%]) persons (Table 1). The highest numbers of cases by race were among White persons (n = 163 [70%]) and by ethnicity were among all non-Hispanic persons (n = 193 [82%]). We did not calculate incidence for race or ethnicity because it was a small dataset, and 25% of data for race and 16% for ethnicity were missing or unknown. The median age of patients was 59 (interquartile range 50–70) years. Most cases occurred among those 50–59 years of age (Table 1). Incidence generally increased with age; the lowest incidence was reported in persons 10–19 and 20–29 years of age (both 0.08 cases/1 million population).

## Discussion

Ehrlichiosis caused by *E. ewingii* is a growing concern in the United States because of increasing geographic distribution and incidence, although cases are likely underreported. When comparing the 2013–2021 and 2008–2012 reporting periods, we observed an ≈2-fold increase in the average number of reported cases during the 2013–2021 period. In addition, during 2013–2021, *E. ewingii* ehrlichiosis was reported from 15 jurisdictions that had not previously reported cases. Although cases increased overall during the reporting period, the number of cases decreased in 2020 and 2021 compared with previous years, likely attributable to the COVID-19 pandemic. Similar trends have been observed for other ehrlichioses and tickborne diseases (*13*). It is unclear if the increase in reported cases is because of improved healthcare provider awareness, availability of molecular testing, increased reporting capabilities, a genuine increase in the number of infected persons, or a combination of those factors.

Reliance on serologic testing to diagnose ehrlichiosis in disease-endemic areas likely contributes to underreporting. During the 2013–2021 period, 56% of *E. chaffeensis* cases reported through NNDSS were classified as probable. Specific laboratory methods for diagnosis are not provided in NNDSS. However, historically, most probable *E. chaffeensis* ehrlichiosis cases have relied on a single serologic result for case classification (*2*). Therefore, it is highly likely that some cases reported as probable *E. chaffeensis* ehrlichiosis were, in fact, *E. ewingii* ehrlichiosis cases, although the proportion is unknown. Reliance on serologic testing could also explain why some states, such as North Carolina, a region with a high incidence of *E. chaffeensis* ehrlichiosis, reported no cases of *E. ewingii* ehrlichiosis. It is possible that healthcare providers in North Carolina were not regularly ordering species-specific PCR testing to identify *E. ewingii* compared with other states.

Molecular testing for *E*. *ewingii* is available at several commercial laboratories in the United States and at CDC. Although healthcare providers should not wait for diagnostic test results before treating patients with suspected ehrlichiosis, PCR can confirm a patient’s illness during the acute phase of infection. PCR provides a rapid confirmation for the healthcare provider and patient and does not require the patient to return for additional serologic testing, saving the patient time and money. In addition to reducing patient costs, improved provider awareness and uptake of PCR-based testing will help define the true burden associated with different *Ehrlichia* spp. In 2024, the ehrlichiosis case definition was revised. The case definition now requires the use of molecular methods to identify and classify specific *Ehrlichia* spp. The 2024 case definition might affect provider testing choices and will likely permit more certainty when describing species-specific cases.

The first limitation of our study is that all case information relied on passive surveillance through NNDSS and not active case finding. A positive *E. ewingii* test result must be reported to public health authorities for investigation and then submitted to NNDSS to be captured in national statistics. If local health departments are unable to investigate cases, those cases would not be reported to NNDSS. Second, cases might be underrepresented because patients had asymptomatic or mild infections. If a person is mildly ill, they might not seek care from their healthcare provider. Thus, cases reported through passive surveillance are more likely to represent patients who are more severely ill and are from disease-endemic areas where healthcare providers have increased awareness of the condition. Finally, results from passive surveillance are not generalizable to the whole population and likely underestimate the true disease burden.

*E. ewingii* infection is an emerging public health concern in the United States. Serologic testing for ehrlichiosis is not species-specific; therefore, molecular testing is essential for diagnosing this disease. Healthcare providers should be aware that PCR testing for ehrlichiosis is highly sensitive and specific during the acute phase of illness. CDC and other partners should also explore novel methods to increase healthcare provider awareness, such as using electronic medical record alerts or working with clinical solutions platforms, such as UpToDate. Novel approaches, along with targeted provider education on *E. ewingii* ehrlichiosis in disease-endemic and emerging regions, could address existing knowledge gaps, improve patient outcomes, and provide a better picture of the epidemiology of this emerging disease.
